# Vicarious Trauma, Rumination, and Vicarious Post-Traumatic Growth: Rethinking the Role of Clinical Supervision

**DOI:** 10.3390/bs16071167

**Published:** 2026-07-10

**Authors:** Sruthi Joy, Shinto Thomas

**Affiliations:** School of Psychological Sciences, CHRIST (Deemed to be University), Dharmaram College Post, Hosur Road, Bengaluru 560029, Karnataka, India; shinto.thomas@christuniversity.in

**Keywords:** rumination, deliberate rumination, clinical supervision, vicarious trauma, vicarious post-traumatic growth, mental health professionals

## Abstract

Mental health professionals are exposed to clients’ traumatic experiences, placing them at risk for vicarious trauma (VT). However, such exposure may also facilitate vicarious post-traumatic growth (VPTG). The present study examined the mediating role of rumination (intrusive and deliberate) in the relationship between VT and VPTG, and the moderating role of clinical supervision. The personal trauma history of the therapist was kept as a covariate. Using a cross-sectional correlational design, data were collected from 278 mental health professionals, including psychologists, counsellors, social workers, and therapists, through purposive sampling. Correlational analyses and moderated mediation analyses were conducted using IBM SPSS. The analysis revealed that VT is positively related to VPTG, and deliberate rumination mediates the relationship between VT and VPTG. Clinical supervision moderates the association between vicarious trauma and deliberate rumination and the association between deliberate rumination and vicarious post-traumatic growth. The findings highlight the role of participation in clinical supervision in shaping these relationships. This study contributes to the limited empirical literature on VT and VPTG and offers implications for clinical supervision practices and professional training.

## 1. Introduction

Trauma exposure can lead to a range of adverse effects, such as depressed mood (Tracy et al., 2014), anxiety, and a reduced capacity for experiencing pleasure (Bonanno, 2004). Although the impact of trauma experience is more on the victim, it can have adverse effects on the individuals who are part of the ecological systems, such as family, caregivers, friends, and helping professionals (Thornton & Perez, 2006). Among these groups, mental health professionals are particularly vulnerable to vicarious trauma, compassion fatigue, and secondary traumatic stress due to the nature of their work, which requires sustained empathic engagement with trauma survivors (McCann & Pearlman, 1990; Figley, 1995).

Repeated vicarious exposure to trauma can negatively impact the individual’s core beliefs about the self, others, and the world. Through sustained empathic attunement to clients’ traumatic narratives, mental health professionals may gradually internalise trauma-related themes, leading to altered perceptions of the world as unsafe, others as untrustworthy, and the self as ineffective or powerless (McCann & Pearlman, 1990). However, recent studies show that vicarious exposure to trauma can also lead to growth termed as vicarious post-traumatic growth. Much like the post-traumatic growth (PTG) experienced by people after direct trauma, these positive changes relate to self-perception, relationships with others and philosophy of life (Abel et al., 2014; Arnold et al., 2005; Brockhouse et al., 2011; Calhoun & Tedeschi, 2013; Manning-Jones et al., 2016; Bartoskova, 2017; Hyatt-Burkhart, 2013; Wheeler & McElvaney, 2018).

Mental health professionals who develop vicarious post-traumatic growth report enhanced therapeutic insight, better empathy, greater professional confidence and a more coherent sense of purpose in their work (Hyatt-Burkhart, 2013). These positive changes can improve clinical judgement, deepen connections with clients, and act as a buffer against occupational distress (Coleman et al., 2018). VPTG may also help improve the clinician’s understanding of human resilience and growth (Ren et al., 2018). In this way, VPTG can function as a protective factor that buffers against burnout and compassion fatigue. However, after experiencing vicarious trauma, not all mental health professionals will experience vicarious post-traumatic growth, as they may remain in a state of distress. Cognitive processes such as rumination play a central role in determining whether the individual will experience vicarious trauma, or they can lead to vicarious post-traumatic growth (Cann et al., 2011). Trauma exposure can disrupt core schemas, prompting rumination as individuals attempt to make sense of the experience. Intrusive rumination tends to maintain distress, whereas deliberate, reflective rumination facilitates meaning-making and schema reconstruction, thereby promoting growth. Without the meaning-making component, which supports growth, professionals may experience persistent intrusive thoughts, reduced emotional responsiveness, diminished professional satisfaction, and poorer work outcomes (K. Cohen & Collens, 2013; Cann et al., 2011). This can reduce the mental health professional’s ability to remain present with clients during therapy and can compromise long-term occupational well-being. Research suggests that several psychological and contextual factors are necessary for growth to occur, such as opportunities for meaning-making, rumination, supportive clinical supervision, and reflective practice.

In this context, clinical supervision may be particularly important. Previous literature has recognised clinical supervision as a foundational element of continuous learning, professional support, and ethical clinical practice. It plays a central role in the training and professional development of mental health practitioners (Borders et al., 2014; Bradley & Becker, 2021). Supervision has the potential to mitigate the adverse effects of VT by facilitating rumination and supporting adaptive cognitive and emotional processing, thereby creating conditions conducive to VPTG. In this way, clinical supervision may function not only as a protective factor but also as a moderating influence on how vicarious trauma is experienced and integrated (Snowdon et al., 2019; Lohani & Sharma, 2023)

Clinical supervision is widely regarded as a positive and necessary component of professional practice; however, within the non-Western mental health care system, the availability of qualified supervisors and the implementation of clinical supervision vary considerably across settings (Pereira & Rekha, 2017). In the absence of standardised supervisory frameworks, supervisory experiences are often shaped by institutional practices, professional seniority, and informal mentorship traditions (Mondal et al., 2021). Although many clinicians report benefiting from clinical supervision, there remains a notable lack of empirical evidence examining how participating in clinical supervision functions specifically in relation to vicarious trauma and vicarious post-traumatic growth among mental health professionals.

Therefore, the present study aims to examine the relationship between vicarious trauma and vicarious post-traumatic growth, with rumination (intrusive and deliberate) as a mediator, and clinical supervision as a moderator. Additionally, personal trauma history is kept as a covariate, given its potential confounding role.

## 2. Theoretical Background and Hypothesis Development

### 2.1. Vicarious Trauma and Vicarious Post-Traumatic Growth

Vicarious trauma (VT) and vicarious post-traumatic growth (VPTG) are related but distinct outcomes of indirect trauma exposure. VT reflects distress and disruptions in core beliefs, whereas VPTG represents positive psychological change resulting from engagement with traumatic material (McCann & Pearlman, 1990; Tedeschi & Calhoun, 1995). Cognitive disruption from VT can catalyse growth, as struggling with distressing material may trigger reflective processing and meaning-making, leading to vicarious post-traumatic growth. Research shows that higher levels of VT can predict greater growth, as the same exposure to trauma that causes vicarious trauma can also inspire growth. Thus, VT can directly lead to VPTG because the cognitive and emotional disruption it creates becomes the foundation for positive change (K. Cohen & Collens, 2013). Hence, we hypothesised that

*There will be a positive relationship between vicarious trauma and vicarious post-traumatic growth*.

### 2.2. Vicarious Trauma and Rumination

Rumination is the catalyst that shapes the aftermath of trauma by either helping the individual toward distress or toward meaning-making and post-traumatic growth (Allbaugh et al., 2016; Cann et al., 2011). Following exposure to traumatic material, individuals often experience cognitive disruption, emotional overwhelm, and a temporary breakdown of core assumptions about the self, others, and the world. Research consistently shows that vicarious trauma can trigger this initial cognitive disturbance, which in turn activates rumination. Research indicates that vicarious trauma can lead to rumination (K. Cohen & Collens, 2013; Long, 2020; Barrington & Shakespeare-Finch, 2013). Traumatic experiences lead to intense cognitive and emotional disruptions, and individuals often engage in rumination to process them. Hence, we hypothesised the following:

*There will be a positive relationship between vicarious trauma and rumination*.

### 2.3. Rumination and Vicarious Post-Traumatic Growth

Among the two types of rumination, previous research has shown that intrusive rumination does not lead to growth. In contrast, deliberate rumination, which mainly deals with meaning-making, helps in forming better schemas about the world after trauma, leading to vicarious post-traumatic growth (Abel et al., 2014; Long, 2020; K. Cohen & Collens, 2013; Dar et al., 2024). Intrusive rumination is associated with poorer outcomes, such as burnout and psychological distress (Cui et al., 2021 Vandevala et al., 2017; Cropley & Zijlstra, 2011). According to post-traumatic theory, deliberate rumination helps individuals to cognitively process the trauma and make new meaning out of their experiences. This cognitive process of meaning-making has been shown to lead to vicarious post-traumatic growth. (Dar et al., 2024; K. Cohen & Collens, 2013; Stockton et al., 2011; Bartoskova, 2017).

*There will be a positive relationship between rumination (Intrusive rumination and deliberate rumination) and vicarious post-traumatic growth*.

### 2.4. Clinical Supervision as a Moderator

Clinical supervision plays a pivotal role in reducing vicarious trauma and fostering vicarious post-traumatic growth among mental health professionals. Research suggests that supervisors often support supervisees through meaning-making processes, aiding in managing VT and facilitating VPTG, even when they are not always aware of the specific coping mechanisms their supervisees employ (Long, 2020). Participating in supervision provides a space for processing trauma exposure and is instrumental in helping practitioners adapt and cope with the emotional toll of their work (Satkunanayagam et al., 2010). Peer and supervisory support, as opposed to organisational support, has a more direct impact on VPTG (Brockhouse et al., 2011).

*Clinical supervision will positively moderate the relationship between vicarious trauma, rumination and vicarious post-traumatic growth*.

### 2.5. Personal Trauma History, Vicarious Trauma and Vicarious Post-Traumatic Growth

Personal trauma history has been shown to influence both vicarious trauma (VT) and vicarious post-traumatic growth (VPTG). Trauma counsellors with personal trauma experiences often report enhanced coping and positive transformations through their work, suggesting that prior trauma may serve as a resilience factor (Jenkins et al., 2011). However, systematic reviews indicate that personal trauma can also increase vulnerability to secondary traumatic stress (Henderson et al., 2024). Mechanistically, personal trauma may disrupt core cognitive schemas about the self, others, and the world, which can be reactivated or reinforced through vicarious exposure to clients’ traumatic experiences (Cleary et al., 2023). Empirical findings further show a positive relationship between VT and VPTG, both of which are associated with personal trauma history (Cosden et al., 2016). These observations highlight the importance of accounting for personal trauma history, as it may independently shape the experience of vicarious trauma and the potential for vicarious post-traumatic growth, beyond the primary mediation or moderation pathways under study.

## 3. Methods

### 3.1. Research Design

The present study adopted a positivist paradigm and followed a cross-sectional correlational design to examine the associations among vicarious trauma, rumination (intrusive and deliberate), clinical supervision and vicarious post-traumatic growth with personal trauma history as a covariate among mental health professionals. This design enabled the identification of patterns and relationships among the study variables at a single point in time using standardised self-report instruments. Initially, correlation analyses were conducted to explore the relationships among the key variables, followed by moderated mediation analysis to test the proposed hypotheses. Data were analysed using PROCESS Macro Model 59 (Hayes, 2018) to test a moderated mediation model. This analytical approach facilitated a comprehensive examination of the complex interplay among vicarious trauma, rumination, clinical supervision, and vicarious post-traumatic growth, thereby providing a nuanced understanding of the mechanisms underlying these relationships.

### 3.2. Participants

The study’s participants predominantly comprise mental health professionals, including clinical psychologists, school psychologists, clinical social workers, counsellors, and therapists drawn from diverse geographic locations within India and working with clients who have experienced trauma. The study adopted purposive sampling and recruited 278 participants. The participants were selected according to the following inclusion criteria: they must have a minimum of a master’s degree, have practised for at least 6 months, see at least 4 clients per week who are survivors of trauma, and be English-speaking. Among the exclusion criteria were foreign nationals working in India, professionals undergoing treatment and medication, and professionals currently not working.

Among the total participants (*N* = 278), 17.3% (*n* = 48) identify as male, 82% (*n* = 228) as female, and 0.7% (*n* = 2) as belonging to other gender categories. In the total sample, 96 participants (34.5%) worked as psychotherapists, 137 (49.3%) as counselling psychologists, 26 (9.4%) as clinical psychologists, 12 (4.3%) as counselling psychologists and teachers/assistant professors, and 7 (2.5%) as PhD scholars and counselling psychologists.

The average years of experience among participants is 5.39, with a standard deviation (SD) of 2.99; the mean age of participants is 29.67, with an SD of 4.31. Clinical Supervision included questions such as “Are you currently undergoing supervision?” Among all participants, 160 reported being currently under supervision. Participants were also asked about how often they seek supervision. Among participants who reported attending, 30.82% attend once a month, 23.27% once a week, 18.24% once every 6 months, 15.09% once every 3 months, 10.06% once every 2 weeks, and 2.52% attend as needed.

### 3.3. Measures

Brief Trauma Questionnaire (BTQ)

It is used to measure personal trauma history. The BTQ is a self-report questionnaire derived from the Brief Trauma Interview (Schnurr et al., 1999). It assesses traumatic exposure according to DSM-IV criteria, focusing on Criterion A (life threat/serious injury). The questionnaire determines whether an individual has had an event that meets the criteria, with exposure scored as positive if the respondent says yes to a life threat or serious injury for specific events. Interrater reliability of BTQ ranges from good to excellent (k = 0.74–1.00), except for life-threatening events (k = 0.60), which is considered acceptable (J. Cohen, 1960).

Vicarious Trauma Scale

The Vicarious Trauma Scale (VTS), developed by Vrklevski and Franklin (2008), measures subjective distress associated with working with traumatised clients. It is an 8-item self-report measure with responses on a 7-point Likert scale. Higher scores indicate more vicarious trauma, with a score range of 0–56 indicating low to high VT. Cronbach’s alpha values range from 0.77 to 0.88, showing high reliability.

Event-Related Rumination Inventory

The Event-Related Rumination Inventory (ERRI) is a 20-item self-report inventory developed by Cann et al. (2011). It includes ten items each for assessing intrusive and deliberate thoughts related to an event, with responses ranging from 0 (not at all) to 3 (often). For this study, instructions are modified to contextualise the scale for trauma experienced through work. For each of the two factors of rumination—intrusive and deliberate rumination—scores range from 0 to 30. Cronbach’s alpha for intrusive rumination items is 0.94, and for deliberate rumination items, it is 0.88.

Post-Traumatic Growth Inventory

The post-traumatic growth inventory—short form (PTGI-SF; Cann et al., 2010) was used to assess VPTG. The PTGI-SF is a 10-item, 6-point Likert-type scale ranging from 0 (I did not experience this change) to 5 (I experienced this change to a very great degree), with a possible total score of 0 to 50. Participants were instructed to focus on their work trauma clients. The wording of response options was altered from “I did not experience/I experienced this change (to a very great degree) as a result of my crisis” to “I did not experience/I experienced this change (to a very great degree) as a result of my work”. While evaluating the underlying factor structure of PTGI-SF, Cann et al. (2010) concluded that a single score can characterise PTGI-SF. In line with this, we tested whether a single PTGI-SF score fits the current data. Cronbach’s alpha in this study was 0.90.

Clinical supervision

Clinical supervision was measured using a single self-report item: “Are you currently undergoing supervision?” Participants responded “Yes” or “No” to indicate whether they were currently receiving professional supervision for their counselling or therapy work. Secondly, they were asked about the frequency of supervision: “How often do you go for supervision?” Participants can respond with “once a week”, “once in two weeks”, “once a month”, “once in three months”, “once in six months”, or “any other specify”.

### 3.4. Procedure

Ethical approval for the work was obtained (RCEC/00617/03/24), and after securing informed consent, a set of questionnaires was distributed to participants to collect data. These questionnaires included a cover letter explaining the purpose of the study, emphasising informed consent and participant confidentiality, and outlining the voluntary nature of participation, with the option to withdraw at any time. Standardised self-report questionnaires were distributed via RedCap, email, and social media. Data were filtered, coded, and analysed using IBM SPSS (version 21.0), in accordance with ethical research guidelines.

## 4. Results

In line with the study’s aim to examine the relationship between vicarious trauma and vicarious post-traumatic growth with rumination as a mediator and supervision as a moderator and personal trauma history as a covariate, data were analysed using IBM SPSS (version 21.0). Correlation analyses were first conducted to explore relationships among the key variables, followed by moderated mediation analysis to test the proposed hypotheses.

As seen in Table 1, there is a significant positive correlation between vicarious post-traumatic growth and vicarious trauma (r = 0.13, *p* < 0.05) and deliberate rumination (0.89, *p* < 0.01). Vicarious post-traumatic growth has a negative relationship with personal trauma history (r = −0.25, *p* < 0.01), as well as with Clinical Supervision (r = −0.48, *p* < 0.01). Notably, vicarious post-traumatic growth does not have an association with intrusive rumination (r = −0.07, *p* = 0.19).

Since there is a high correlation between deliberate rumination and vicarious post-traumatic growth, the data were screened for multicollinearity. Multicollinearity occurs when there is a high correlation between the independent variables (Hair et al., 2010; Tabachnick & Fidell, 2013). Kline (2005) suggests that bivariate correlations of r = 0.85 or greater indicate multicollinearity. In the present study, multicollinearity was assessed using tolerance and variance inflation factor (VIF) values. The tolerance values ranged from 0.646 to 0.942, which is above the threshold of 0.10, and the VIF values ranged from 1.062 to 1.548, which is below the threshold of 10 (Field, 2013; Hair et al., 2010). Therefore, there is no evidence of multicollinearity among the predictor variables. To assess the potential influence of common method variance, Harman’s single-factor test was conducted. An unrotated principal component analysis revealed 41.20% of the total variance, which is below the recommended threshold of 50% (Podsakoff et al., 2003). These findings suggest that common method variance is unlikely to have substantially influenced the results.

### Moderated Mediation Analysis

Moderated Mediation analyses were conducted to examine whether the indirect effects of vicarious trauma on vicarious post-traumatic growth were mediated by rumination (intrusive and deliberate), varied across the presence or absence of supervision, while controlling for personal trauma history as presented in Table 2 and Table 3.

Moderated Mediation with Intrusive rumination as mediator

**Figure 1:**
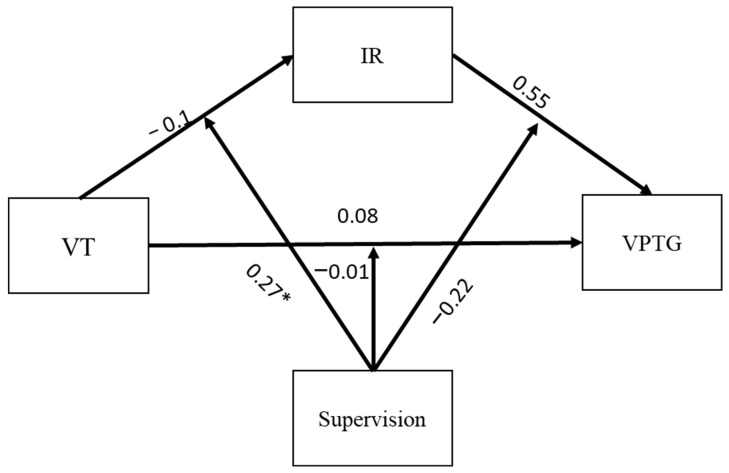
Intrusive rumination as the mediator and Clinical Supervision as the moderator (Note. * Indicates a statistically significant path (*p* < 0.05).

Vicarious trauma did not show any statistically significant direct effect on vicarious post-traumatic growth (*b* = 0.08, *t* = 0.41, *p* = 0.68) or on intrusive rumination (Mediator) (*b* = −0.10, *t* = −0.83, *p* = 0.41). Intrusive rumination did not show any direct effect on vicarious post-traumatic growth (*b* = 0.55, *t* = 1.88, *p* = 0.06). The pathway effect of vicarious trauma and intrusive rumination on vicarious post-traumatic growth is not significant at either level of supervision, as seen in Table 3**.** When supervision was present (coded = 1), the indirect effect was *b* = 0.06, Boot *SE* = 0.04, 95% CI [0.00, 0.15]. When supervision was not present (coded = 2), the indirect effect was *b* = 0.05, Boot *SE* = 0.11, 95% CI [−0.21, 0.25]. Therefore, intrusive rumination does not mediate the relationship between vicarious trauma and vicarious post-traumatic growth. Though supervision does not predict intrusive rumination (*b* = −3.73, *t* = −1.49, *p* = 0.14), it does have a significant moderating effect on intrusive rumination (*b* = 0.27, *t* = 3.74, *p* < 0.001). Indicating that the effect of VT on intrusive rumination varied depending on the presence of supervision. Supervision does not moderate the relationship between vicarious trauma and vicarious post-traumatic growth (*b* = −0.01, *t* = −0.09, *p* = 0.93) and also between intrusive rumination and vicarious post-traumatic growth (*b* = −0.22, *t* = −1.09, *p* = 0.28). See Figure 1 for a visual representation. The covariate personal trauma history shows a direct effect on vicarious post-traumatic growth (*b* = −1.48, t = −3.60, *p* = 0.00). The index of moderated mediation was not significant, index = −0.01, Boot *SE* = 0.12, 95% CI [−0.28, 0.21], indicating that the indirect effect of VT on VPTG via intrusive rumination did not differ significantly across levels of supervision.

Moderated Mediation with Deliberate rumination as mediator

**Figure 2:**
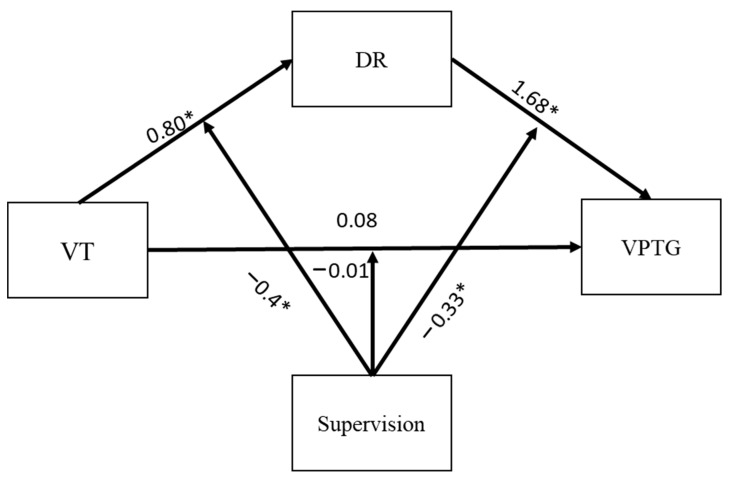
Deliberate rumination as the mediator and Clinical Supervision as the moderator. (Note. * Indicates a statistically significant path (*p* < 0.05).

As seen in Table 4 Vicarious trauma does not show a statistically significant direct effect on vicarious post-traumatic growth (*b* = 0.08, *t* = 0.41, *p* = 0.68). Vicarious trauma has a significant effect on deliberate rumination (Mediator) (*b* = 0.80, *t* = 3.52, *p* = 0.00). Deliberate rumination shows a direct effect on vicarious post-traumatic growth (*b* = 1.68, *t* = 11.48, *p* = 0.00). The indirect effect of VT on VPTG via DR was conditional on supervision (Table 3). When supervision was present (coded = 1), the indirect effect was significant, *b* = 0.55, Boot *SE* = 0.17, 95% Boot CI [0.21, 0.88]. When supervision was not present (coded = 2), the indirect effect was not significant, *b* = 0.01, Boot *SE* = 0.05, 95% Boot CI [−0.09, 0.10]. Clinical Supervision has a significant moderating effect on vicarious trauma and deliberate rumination (*b* = −40, *t* = −3.15, *p* = 0.00). Similarly, Clinical Supervision moderates the relationship between deliberate rumination and vicarious post-traumatic growth (*b* = −0.33, *t* = −3.14, *p* = 0.00). However, vicarious trauma and Clinical Supervision (*b* = −0.01, *t* = −0.09, *p* = 0.93) did not show an interaction effect on vicarious post-traumatic growth. The covariate personal trauma history shows a direct effect on vicarious post-traumatic growth (*b* = −1.48, *t* = −3.60, *p* = 0.00). See Figure 2 for a visual representation. The index of moderated mediation confirmed that this conditional indirect effect was statistically significant, index = −0.54, Boot *SE* = 0.18, 95% Boot CI [−0.87, −0.19], indicating that supervision significantly attenuated the mediating effect of DR.

## 5. Discussion

The present study explored the relationship between vicarious trauma and vicarious post-traumatic growth among mental health professionals. Specifically focusing on the mediating role of intrusive and deliberate rumination on the relationship between vicarious trauma and vicarious post-traumatic growth, and the moderating role of Clinical Supervision. The analysis revealed that deliberate rumination statistically mediates the relationship between vicarious trauma and vicarious post-traumatic growth. Clinical Supervision moderates the relationship between vicarious trauma and deliberate rumination, and deliberate rumination and vicarious post-traumatic growth.

In the present study, results from correlation analysis show that there is a relationship between vicarious trauma and vicarious post-traumatic growth. This indicates that exposure to clients’ traumatic experiences may be linked with distress and can lead to growth. Previous literature has shown that vicarious trauma can lead to disruption of core beliefs. This may create the conditions necessary for meaning-making and vicarious post-traumatic growth (Brockhouse et al., 2011). These findings confirm that cognitive engagement after experiencing vicarious trauma may not be uniformly adaptive. Rather, the quality and intentionality of rumination are critical factors in determining the path from distress to growth.

Deliberate rumination is positively and significantly correlated with VPTG. This aligns with the Post-Traumatic Growth Theory (Tedeschi & Calhoun, 2004), which identifies that intentional and reflective cognitive processing is a central mechanism by which trauma-exposed individuals reconstruct shattered worldviews into more complex and integrated meaning systems. Professionals who engage meaningfully with trauma-related experiences, reflecting on their implications and re-evaluating their assumptions about themselves and the world, are more likely to experience growth-related outcomes (Taku et al., 2009).

Notably, intrusive rumination, which is automatic and repetitive, was positively associated with VT but not with VPTG. Consistent with previous literature, uncontrolled cognitive intrusions are predominantly distress-maintaining rather than growth-facilitating (Cann et al., 2011). The results reaffirm that not all forms of cognitive engagement are helpful; deliberate rumination is adaptive, while intrusive rumination may exacerbate distress.

Clinical supervision showed a negative association with both deliberate rumination and VPTG. This pattern, while counterintuitive given prevailing assumptions about the protective function of supervision, is discussed in detail in the moderation section below.

### 5.1. The Mediating Role of Intrusive and Deliberate Rumination

In the present study, intrusive rumination does not mediate the relationship between vicarious trauma and vicarious post-traumatic growth. Although intrusive rumination was positively associated with vicarious trauma, it did not mediate vicarious post-traumatic growth. Intrusive rumination involves a passive, uncontrollable, and unwanted focus on the causes and consequences of distress. This suggests that automatic, unwanted, and distress-focused cognitive processing may contribute to psychological strain (Taku et al., 2009). However, it is not sufficient to facilitate the positive psychological change required for vicarious post-traumatic growth. Overall, the findings indicate that intrusive rumination is more closely related to vicarious trauma. In contrast to intrusive rumination, deliberate rumination emerged as a more adaptive cognitive process in relation to vicarious trauma and post-traumatic growth.

In the current study, deliberate rumination mediates the relationship between vicarious trauma and vicarious post-traumatic growth. This aligns with the post-traumatic growth theory (Tedeschi & Calhoun, 2004), which holds that deliberate rumination helps individuals cognitively process trauma and make new meaning out of their experiences, fostering growth.

The mediating role of deliberate rumination is particularly meaningful in the context of mental health professionals. They are required to engage in repeated reflection on trauma-related material, both within and outside of clinical encounters, as part of their work. When this engagement is intentional and purposeful, rather than involuntary or distress-driven, it appears to enable the cognitive restructuring and meaning reconstruction that underpin VPTG (Taku et al., 2009). These findings thus extend prior research on post-traumatic growth in direct trauma survivors to the vicarious trauma context, demonstrating that the same reflective mechanisms operate when trauma is experienced indirectly through clinical work. For mental health professionals, cultivating the capacity for deliberate, structured reflection on vicarious trauma experiences may therefore be a critical component of professional resilience and growth.

### 5.2. The Moderating Role of Clinical Supervision

Clinical supervision significantly moderated the relationship between vicarious trauma and intrusive rumination, as indicated by the significant interaction between vicarious trauma and supervision. Although the interaction accounted for a small proportion of variance in intrusive rumination, the findings suggest that supervision may encourage greater reflection on professionals’ experiences of clients’ trauma (Satkunanayagam et al., 2010). This may lead to more intrusive thoughts rather than the immediate relief from distress. From the perspective of post-traumatic growth theory (Tedeschi & Calhoun, 2004), this represents an important phase as it may facilitate meaning-making and deliberate rumination.

When deliberate rumination was examined as a mediator, the moderated mediation analysis indicated that clinical supervision moderated the relationship between vicarious trauma and deliberate rumination and between the association of deliberate rumination and vicarious post-traumatic growth. Although the interaction effect was small, the moderated mediation analysis revealed a significant conditional indirect effect through deliberate rumination. The indirect effect of vicarious trauma on vicarious post-traumatic growth was evident in the group who were participants in supervision compared to the professionals who were not. This indicates that supervision altered the role of deliberate rumination in the growth process. In contrast, no significant moderated mediation was observed through intrusive rumination, suggesting that supervision did not meaningfully influence the indirect pathway involving distress-oriented cognitive processing.

These findings are contrary to existing assumptions in the supervision literature, which broadly characterise clinical supervision as a facilitative and protective resource that supports professional wellbeing and reflective practice (Rothwell et al., 2021; Baldiwala et al., 2022).

One theoretically coherent explanation for this pattern is that the average years of experience in the present sample were 5.3 years, with the majority of participants having 5 years or fewer of clinical experience, placing them within the novice-to-early-career stage of professional development, which is considered a novice therapist. For novice MHPs, clinical supervision prioritises case formulation, therapist-led risk management, and procedural competence (Rønnestad et al., 2025). Particularly in high-pressure or resource-constrained settings, this may be insufficient to address the supervisee’s subjective experience of vicarious trauma (Mondal et al., 2021). When clinical supervision remains predominantly case-focused rather than person-focused, opportunities for deliberate rumination and meaning reconstruction may be limited, constraining pathways to VPTG (Rothwell et al., 2021).

Another possible reason is that supervision frequency in the sample varies. This might also mean that supervisory exposure is in a continuum rather than a single intervention. Therefore, the observed differences may reflect the intensity and regularity of supervisory contact. More frequent supervision is likely to provide more consistent opportunities for reflective practice, stress management, and learning. In contrast, infrequent supervision may be more limited in scope. Accordingly, the moderating effect observed in the present study may reflect differences in the frequency and continuity of supervisory contact rather than supervision as a uniform or standardised construct, highlighting the variability in how supervision is experienced and operationalised across settings (Choy-Brown & Stanhope, 2018).

In the present study. Clinical supervision was measured using a single dichotomous item indicating whether participants were currently receiving supervision; the study was unable to assess important characteristics of supervision, including its quality, duration, format, theoretical orientation, and primary focus. As a result, the observed moderation effect may reflect unmeasured variations in the nature and purpose of supervision rather than the influence of supervision itself. Therefore, the findings should not be interpreted as evidence that clinical supervision weakens growth-related processes, but rather as indicating that different supervisory experiences may be associated with different patterns of trauma processing and growth.

Another important consideration is the inherently personal nature of vicarious post-traumatic growth. Growth appears to require active ownership of one’s meaning-making process, in which professionals integrate vicarious trauma experiences into their broader sense of self, values, and professional identity. Clinical Supervision may facilitate this process but cannot substitute for the internal cognitive and emotional work necessary for growth (Rothwell et al., 2021). Moreover, professionals who actively seek or require clinical supervision may disproportionately be those who are currently experiencing higher levels of distress, emotional burden, or professional uncertainty. In such cases, clinical supervision may function as a stabilising intervention during an ongoing struggle, rather than as a context in which growth has already begun to emerge. In such cases, clinical supervision may serve as a support mechanism during ongoing struggle, rather than directly facilitating growth itself.

### 5.3. Implications

The present study extends Post-Traumatic Growth Theory (Tedeschi & Calhoun, 2004) to the vicarious trauma context and to non-Western professional populations, empirically establishing deliberate rumination as a mediating mechanism in the VT–VPTG pathway. The moderated mediation model further identifies clinical supervision as a factor that can constrain, rather than facilitate, growth-oriented processing under certain contextual and developmental conditions. Collectively, these findings call for more nuanced theoretical models of VPTG that are sensitive to interactions among reflective processes, supervisory contexts, and cross-cultural variation in how supervision is structured and experienced.

Across international mental health settings, clinical supervision is predominantly structured around clinical accountability, risk management, and competency development, with limited formal attention to the supervisee’s subjective experience of vicarious trauma (Rothwell et al., 2021; Mondal et al., 2021). Given the strong association between deliberate rumination and VPTG observed in this study, professional training and support systems may benefit from creating opportunities for structured reflection on trauma-related clinical work. Reflective practices such as journalling, peer consultation, case reflection, and supervision that encourage discussion of personal and professional responses to exposure to trauma may support meaning-making processes associated with growth. Organisations may also consider incorporating reflective and trauma-informed elements into existing support structures to help clinicians process the emotional impact of their work.

The findings from the current study carry particular salience for mental health systems in low- and middle-income countries (LMICs), where high caseloads, lack of regulation on supervision, and cultural norms discouraging emotional disclosure increase the burden of vicarious trauma (Chakrapani & Bharat, 2023). Where supervision exists, it often prioritises the case over reflective practice and does not focus on meaning-making. However, in such low and middle-income countries, there is a heightened need for deliberate rumination. This suggests that concentrating on reflective supervisory practice even within constrained systems could yield meaningful gains in practitioner wellbeing and workforce sustainability. The critical policy question for LMIC settings is not merely whether supervision exists, but whether it is intentionally designed to support the growth, and not only the clinical competence, of the professionals who deliver care.

### 5.4. Future Research

Future research should adopt more comprehensive and methodologically robust approaches to better understand the relationships among vicarious trauma, rumination, supervision, and vicarious post-traumatic growth. In particular, studies should assess supervision as a multidimensional construct by examining its quality, frequency, duration, format, theoretical orientation, supervisory alliance, and functional focus. This would help clarify how different supervisory experiences are associated with cognitive processing and growth-related outcomes. Longitudinal designs are particularly needed to capture the temporal dynamics of these processes and to examine how clinical supervision functions across distinct career stages. Early-career professionals, for instance, may depend on clinical supervision for containment and emotional validation, whereas more experienced practitioners may engage in deeper meaning-making and identity transformation. Examining these developmental trajectories would help in advancing understanding of clinical supervision not merely as a protective resource, but as an active developmental context that shapes trauma-related adaptation over time (Duggal & Rao, 2016). Additionally, future research should consider potential contextual and organisational factors that could also influence, such as coping style, workload, trauma caseload, and institutional support, which may influence both supervision engagement and trauma-related outcomes. Furthermore, research should examine role-specific differences among mental health professionals. Varying clinical responsibilities and levels of exposure to trauma may differentially influence vicarious trauma, vicarious post-traumatic growth, and the effectiveness of supervision (Gesi et al., 2023). Such analyses would provide a more nuanced understanding of occupation-specific risk and protective factors. Examining these factors together would provide a more nuanced understanding of supervision not merely as a protective factor, but as a dynamic professional context that may shape both distress and growth among mental health professionals.

## 6. Limitations and Conclusions

Several limitations should be acknowledged. First, the cross-sectional design precludes any conclusions regarding temporal ordering or causality. Although the proposed model was theoretically informed, the observed associations should be interpreted as statistical relationships rather than evidence of directional or developmental processes. Second, all variables were assessed using self-report measures, which may introduce common-method variance. This is particularly relevant given the strong association observed between deliberate rumination and vicarious post-traumatic growth. Future research should examine the discriminant validity of these constructs using multi-method and longitudinal designs. Third, clinical supervision was measured using a single dichotomous item (yes/no), which does not capture its multidimensional nature. Important aspects such as quality, frequency, duration, format, theoretical orientation, supervisory alliance, and functional focus were not assessed. Consequently, the findings reflect differences between participants who reported receiving supervision and those who did not, rather than the effects of specific supervisory practices. Fourth, although personal trauma history was statistically controlled, several potentially relevant confounding variables were not assessed. These include coping styles, organisational support, workload, type of trauma clients, trauma caseload, institutional culture, and severity of client trauma exposure. The absence of these variables limits the ability to fully interpret the observed relationships, particularly those involving clinical supervision. Fifth, the sample consisted of mental health professionals working in India. While this provides important context-specific insight, it may limit the generalisability of the findings to other cultural and organisational contexts where supervision structures and professional practices may differ. Future studies should allow for a more nuanced understanding of how these processes evolve.

In conclusion, the present study highlights the complex relationship between vicarious trauma (VT) and vicarious post-traumatic growth (VPTG) among mental health professionals. The findings indicate that VT and VPTG can exist on a continuum, suggesting that exposure to clients’ traumatic experiences may lead not only to distress but also to opportunities for psychological growth. A key finding of the study is the mediating role of deliberate rumination. It is the intentional, reflective thinking about clients’ trauma-related experiences that can help professionals construct new meaning, thereby facilitating growth. In contrast, intrusive rumination was associated with higher levels of VT but did not contribute to VPTG, highlighting that not all cognitive engagement with trauma leads to positive outcomes. Clinical supervision appeared to play a complex and context-dependent role in the present study. The moderated mediation results indicated that supervision was associated with weaker relationships between vicarious trauma, deliberate rumination, and vicarious post-traumatic growth. Moreover, Supervision moderated the relationship between vicarious trauma and intrusive rumination. However, given that supervision was assessed using a single dichotomous indicator (yes/no), these findings should be interpreted with caution. It is not possible to determine whether these differences reflect the presence of supervision itself or unmeasured variations in its quality, content, purpose, or the broader professional context in which it occurs. The capacity of supervision to support reflective processing and meaning-making may therefore vary depending on how it is structured and experienced in practice. In addition, structural and organisational factors such as workload, caseload intensity, and access to supervisory support may also shape how professionals process vicarious trauma and engage in deliberate rumination. Overall, the findings suggest that deliberate rumination is associated with vicarious post-traumatic growth. At the same time, supervisory and contextual factors may be related to differences in these associations, warranting further investigation using more detailed and longitudinal designs.

## Figures and Tables

**Table 1:** The relationship between study variables.

Variable	VT	IR	DR	BTQ	CP
IR	0.54 **				
DR	0.07	−0.08			
BTQ	−0.01	0.21 **	−0.20 **		
CP	0.05	0.55 **	−0.46 **	0.12 **	
VPTG	0.13 *	−0.07	0.89 **	−0.25 **	−0.48 **

* *p* < 0.05. ** *p* < 0.01. Note: VT = Vicarious Trauma; IR = Intrusive Rumination; DR = Deliberate Rumination; VPTG = Posttraumatic Growth Inventory; BTQ = Brief Trauma Questionnaire; CP = Clinical Supervision.

**Table 2:** Direct and interaction effects of clinical supervision as the moderator and intrusive rumination as the mediator.

Model Pathway	*b*	*SE*	*t*	*p*	Bias-Corrected Bootstrap 95% CI	
					LL 95% CI	UL 95% CI
*Direct Effect*						
Vicarious trauma → Intrusive rumination	−0.1	0.12	−0.83	0.41	−0.33	0.13
Supervision → Intrusive Rumination	−3.73	2.49	−1.49	0.14	−8.64	1.18
Vicarious trauma → Vicarious post-traumatic growth	0.08	0.19	0.41	0.68	−0.3	0.46
Intrusive rumination → Vicarious post-traumatic growth	0.55	0.3	1.88	0.06	−0.03	1.14
*Interaction effect*						
Vicarious trauma × Supervision → intrusive rumination	0.27	0.06	4.18	0	0.14	0.4
Vicarious trauma × Supervision → Vicarious post-traumatic growth	−0.01	0.12	−0.09	0.93	−0.25	0.23
Intrusive Rumination × Supervision → Vicarious post-traumatic growth	−0.22	0.21	−1.09	0.28	−0.63	0.18

**Table 3:** Conditional Indirect Effects.

Indirect Path	Supervision	Effect	Boot *SE*	Bias-Corrected Bootstrap 95% CI	
				LL 95% CI	UL 95% CI
VT → IR → VPTG	1	0.06	0.04	0	0.15
VT → IR → VPTG	2	0.05	0.11	−0.21	0.25
VT → DR → VPTG	1	0.55	0.17	0.2	0.88
VT → DR → VPTG	2	0.01	0.05	−0.1	0.1

Note. VT = vicarious trauma; IR = intrusive rumination; DR = deliberate rumination; VPTG = vicarious post-traumatic growth. Supervision values represent those currently receiving clinical supervision (1) and those not receiving it (2). Indirect effects were estimated using bootstrapping (5000 resamples), and bias-corrected bootstrap confidence intervals (95%) are reported.

**Table 4:** Direct and interaction effect of clinical supervision as the moderator and deliberate rumination as the mediator.

Model Pathway	*b*	*SE*	*t*	*p*	Bias-Corrected Bootstrap 95% CI	
					LL 95% CI	UL 95% CI
*Direct Effect*						
Vicarious trauma → Deliberate rumination	0.8	0.23	3.52	0	0.35	1.25
Clinical Supervision → Deliberate rumination	6.83	4.87	1.4	0.16	−2.76	16.42
Vicarious trauma → Vicarious post-traumatic growth	0.08	0.19	0.41	0.68	−0.3	0.46
Deliberate rumination → Vicarious post-traumatic growth	1.68	0.15	11.48	0	1.39	1.97
*Interaction effect*						
Vicarious trauma × clinical supervision → Deliberate rumination	−0.4	0.13	−3.15	0	−0.64	−0.15
Vicarious trauma × clinical supervision taking → Vicarious post-traumatic growth	−0.01	0.12	−0.09	0.93	−0.25	0.23
Deliberate Rumination × clinical supervision → Vicarious post-traumatic growth	−0.33	0.11	−3.14	0	−0.54	−0.12

## Data Availability

The data presented in this study are available upon request from the corresponding author due to privacy and confidentiality restrictions regarding participant information.

## Abbreviations

The following abbreviations are used in this manuscript:

VT	Vicarious trauma
VPTG	Vicarious post-traumatic growth
IR	Intrusive rumination
DR	Deliberate rumination
